# The genome sequence of a dung beetle, *Geotrupes spiniger *(Marsham, 1802)

**DOI:** 10.12688/wellcomeopenres.23705.1

**Published:** 2025-02-13

**Authors:** František Sládeček, Owen T. Lewis, University of Oxford and Wytham Woods Genome Acquisition Lab, Darwin Tree of Life Barcoding collective, Wellcome Sanger Institute Tree of Life Management, Samples and Laboratory team, Wellcome Sanger Institute Scientific Operations: Sequencing Operations, Wellcome Sanger Institute Tree of Life Core Informatics team, Tree of Life Core Informatics collective, Darwin Tree of Life Consortium

**Affiliations:** 1 Department of Zoology, Faculty of Science, University of South Bohemia, České Budějovice, Czech Republic; 2 Department of Biology, University of Oxford, Oxford, England, UK

**Keywords:** Geotrupes spiniger, dung beetle, genome sequence, chromosomal, Coleoptera

## Abstract

We present a genome assembly from an individual male specimen of the dung beetle, *Geotrupes spiniger* (Arthropoda; Insecta; Coleoptera; Geotrupidae). The genome sequence has a total length of 580.60 megabases. Most of the assembly (81.94%) is scaffolded into 12 chromosomal pseudomolecules, including the X and Y sex chromosomes. The mitochondrial genome has also been assembled and is 21.66 kilobases in length. Gene annotation of this assembly on Ensembl identified 12,820 protein-coding genes.

## Species taxonomy

Eukaryota; Opisthokonta; Metazoa; Eumetazoa; Bilateria; Protostomia; Ecdysozoa; Panarthropoda; Arthropoda; Mandibulata; Pancrustacea; Hexapoda; Insecta; Dicondylia; Pterygota; Neoptera; Endopterygota; Coleoptera; Polyphaga; Scarabaeiformia; Scarabaeoidea; Geotrupidae; *Geotrupes*; *Geotrupes spiniger* (Marsham, 1802) (NCBI:txid295525)

## Background

*Geotrupes spiniger* is an economically important dung beetle in the family Geotrupidae. It is large-bodied (up to 25 mm in length) and the body is shiny and black on the dorsal surface, with a metallic blue/purple underside.

*Geotrupes spiniger* is native to Europe eastwards to Ukraine and the Middle East. It also has a non-native distribution in southeastern Australia and in New Zealand ( GBIF Secretariat, 2023), where *G. spiniger* has been introduced alongside other dung beetles to provide ecosystem services associated with the processing and burial of livestock dung, since native dung beetles are unable to remove the large quantities of dung produced by introduced domestic livestock ( Forgie *et al.*, 2018).

In Britain and Ireland, *G. spiniger* (and the closely-related and morphologically very similar *G. stercorarius*) are among the largest dung beetle species and it has a wide distribution, although most records are from the southern half of Britain ( NBN Atlas Partnership, 2025). Adults are especially active at dusk and dawn; they are attracted to light and are often caught in moth traps.

*Geotrupes spiniger* breeds by digging tunnels up to 45 cm in length below the dung of large herbivores such as cattle and horses. From these vertical tunnels it creates a series of horizontal chambers which are provisioned with sausage-shaped dung masses ( Klemperer, 1979), with one egg laid in each dung mass. Research on the biology of *G. spiniger* includes studies of antennal responses to volatiles ( Perera *et al.*, 2023), sensitivity to insecticides ( Manning *et al.*, 2018), and the use of cuticular hydrocarbons to investigate taxonomy and phylogeny ( Niogret *et al.*, 2019).

**Figure 1.**
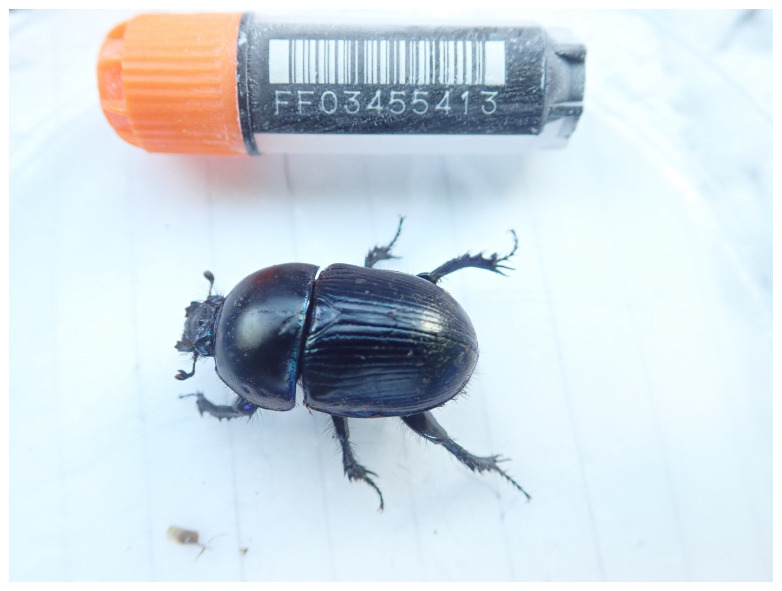
Photograph of the *Geotrupes spiniger* (icGeoSpin1) specimen used for genome sequencing.

The genome of *Geotrupes spiniger* was sequenced as part of the Darwin Tree of Life Project, a collaborative effort to sequence all named eukaryotic species in the Atlantic Archipelago of Britain and Ireland. Here we present a chromosomally complete genome sequence for *Geotrupes spiniger*, based on a male specimen from Wytham Farm, United Kingdom ( Figure 1).

## Genome sequence report

The genome of *Geotrupes spiniger* ( Figure 1) was sequenced using Pacific Biosciences single-molecule HiFi long reads, generating a total of 45.71 Gb (gigabases) from 4.86 million reads, providing an estimated 86-fold coverage. Chromosome conformation Hi-C sequencing produced 101.43 Gb from 671.71 million reads. Specimen and sequencing details are summarised in Table 1.

**Table 1.** Specimen and sequencing data for *Geotrupes spiniger*.

Project information			
**Study title**	Geotrupes spiniger		
**Umbrella BioProject**	PRJEB64062		
**Species**	*Geotrupes spiniger*		
**BioSample**	SAMEA7520022		
**NCBI taxonomy ID**	295525		
Specimen information			
**Technology**	**ToLID**	**BioSample** **accession**	**Organism** **part**
**PacBio long read sequencing**	icGeoSpin1	SAMEA7520299	thorax
**Hi-C sequencing**	icGeoSpin1	SAMEA7520100	head
**RNA sequencing**	icGeoSpin1	SAMEA7520299	thorax
Sequencing information			
**Platform**	**Run accession**	**Read count**	**Base count** **(Gb)**
**Hi-C Illumina NovaSeq 6000**	ERR11679369	6.72e+08	101.43
**PacBio Sequel IIe**	ERR11673226	2.48e+06	24.04
**PacBio Sequel IIe**	ERR11673225	2.38e+06	21.67
**RNA Illumina NovaSeq 6000**	ERR11837498	7.75e+07	11.7
**RNA Illumina NovaSeq 6000**	ERR11837497	7.17e+07	10.82

Assembly errors were corrected by manual curation, including 7 missing joins or mis-joins and one haplotypic duplication. This reduced the assembly length by 1.57% and the scaffold number by 1.63%, and increased the scaffold N50 by 2.58%. The final assembly has a total length of 580.60 Mb in 180 sequence scaffolds, with 79 gaps, and a scaffold N50 of 44.0 Mb ( Table 2).

**Table 2.** Genome assembly data for *Geotrupes spiniger*, icGeoSpin1.1.

Genome assembly		
Assembly name	icGeoSpin1.1	
Assembly accession	GCA_959613385.1	
*Accession of alternate haplotype*	*GCA_959613335.1*	
Span (Mb)	580.60	
Number of contigs	260	
Number of scaffolds	180	
Longest scaffold (Mb)	65.61	
Assembly metrics *		*Benchmark*
Contig N50 length (Mb)	7.3	*≥ 1 Mb*
Scaffold N50 length (Mb)	44.0	*= chromosome N50*
Consensus quality (QV)	62.0	*≥ 40*
*k*-mer completeness	100.0%	*≥ 95%*
BUSCO v5.4.3 lineage: endopterygota_odb10	C:99.5%[S:98.7%,D:0.8%], F:0.1%,M:0.4%,n:2,124	*S > 90%, D < 5%*
Percentage of assembly mapped to chromosomes	81.94%	*≥ 90%*
Sex chromosomes	XY	*localised homologous pairs*
Organelles	Mitochondrial genome: 21.66 kb	*complete single alleles*
Genome annotation of assembly GCA_959613385.1 at Ensembl		
Number of protein-coding genes	12,820	
Number of non-coding genes	1,582	
Number of gene transcripts	21,852	

* Assembly metric benchmarks are adapted from Rhie *et al.* (2021) and the Earth BioGenome Project Report on Assembly Standards September 2024. ** BUSCO scores based on the endopterygota_odb10 BUSCO set using version 5.4.3. C = complete [S = single copy, D = duplicated], F = fragmented, M = missing, n = number of orthologues in comparison. A full set of BUSCO scores is available at https://blobtoolkit.genomehubs.org/view/icGeoSpin1_1/dataset/icGeoSpin1_1/busco.

**Figure 2.**
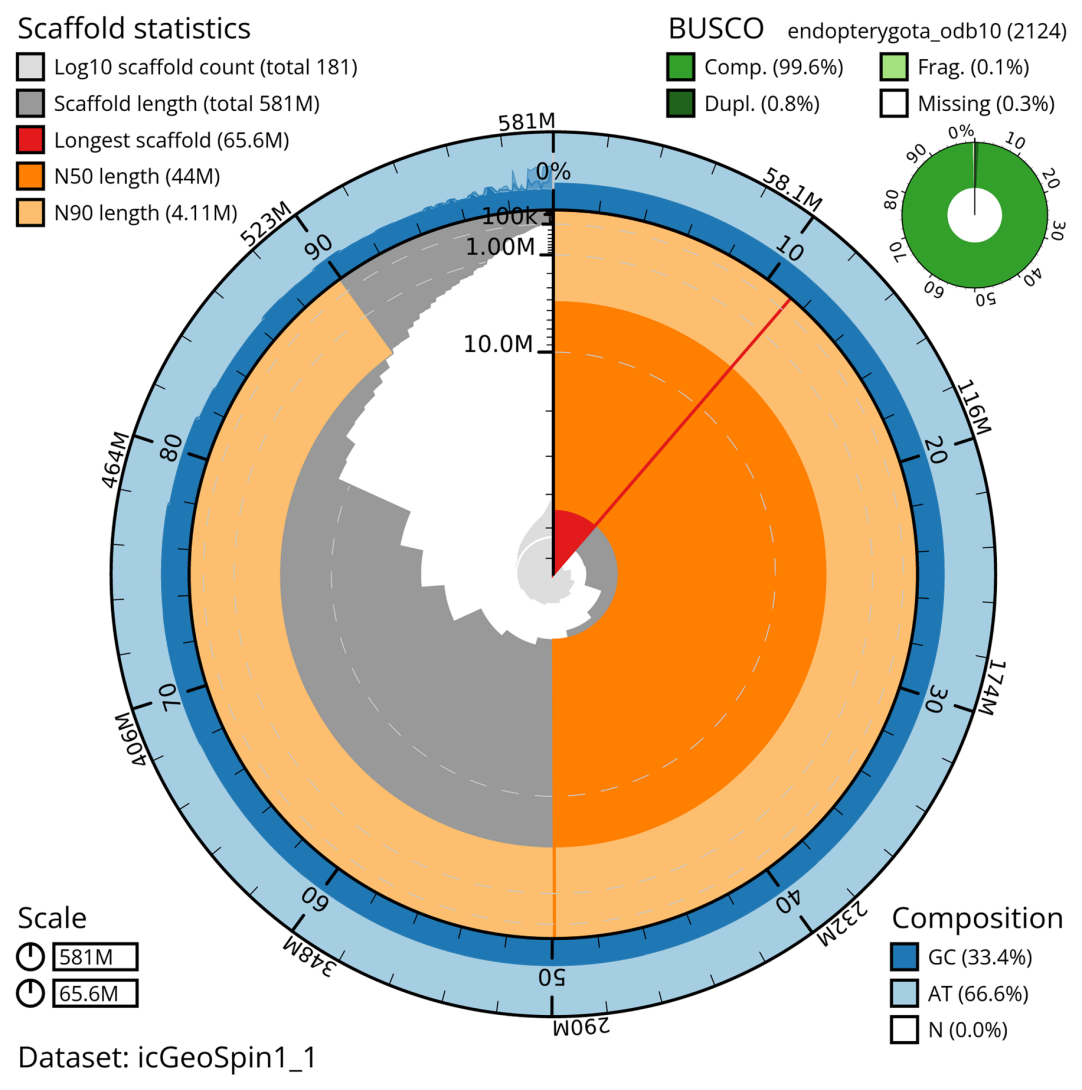
Genome assembly of *Geotrupes spiniger*, icGeoSpin1.1: metrics. The BlobToolKit snail plot provides an overview of assembly metrics and BUSCO gene completeness. The circumference represents the length of the whole genome sequence, and the main plot is divided into 1,000 bins around the circumference. The outermost blue tracks display the distribution of GC, AT, and N percentages across the bins. Scaffolds are arranged clockwise from longest to shortest and are depicted in dark grey. The longest scaffold is indicated by the red arc, and the deeper orange and pale orange arcs represent the N50 and N90 lengths. A light grey spiral at the centre shows the cumulative scaffold count on a logarithmic scale. A summary of complete, fragmented, duplicated, and missing BUSCO genes in the endopterygota_odb10 set is presented at the top right. An interactive version of this figure is available at https://blobtoolkit.genomehubs.org/view/icGeoSpin1_1/dataset/icGeoSpin1_1/snail.

**Figure 3.**
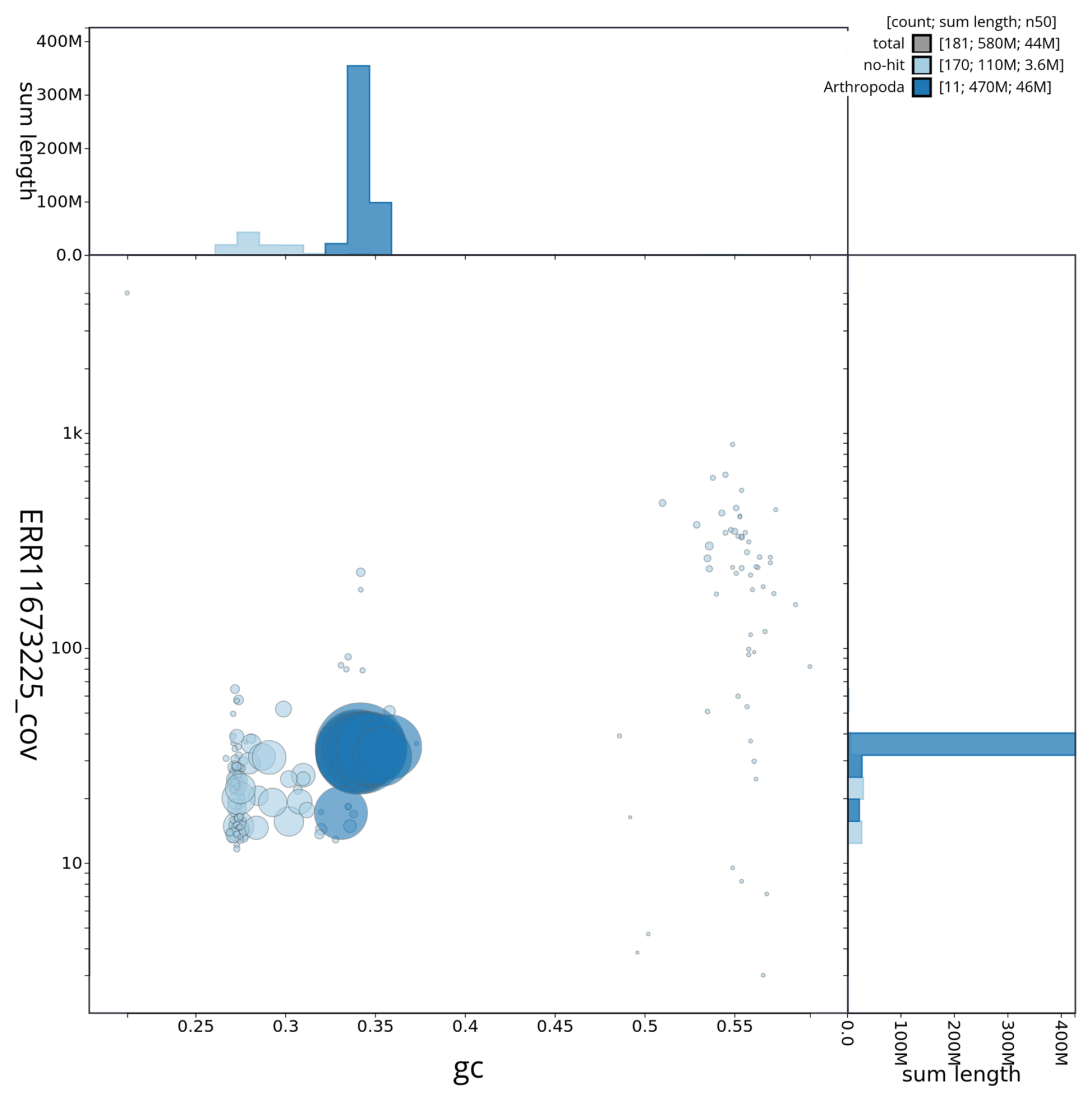
Genome assembly of *Geotrupes spiniger*, icGeoSpin1.1: BlobToolKit GC-coverage plot. BlobToolKit GC-coverage plot showing sequence coverage (vertical axis) and GC content (horizontal axis). The circles represent scaffolds, with the size proportional to scaffold length and the colour representing phylum membership. The histograms along the axes display the total length of sequences distributed across different levels of coverage and GC content. An interactive version of this figure is available at https://blobtoolkit.genomehubs.org/view/icGeoSpin1_1/dataset/icGeoSpin1_1/blob.

**Figure 4.**
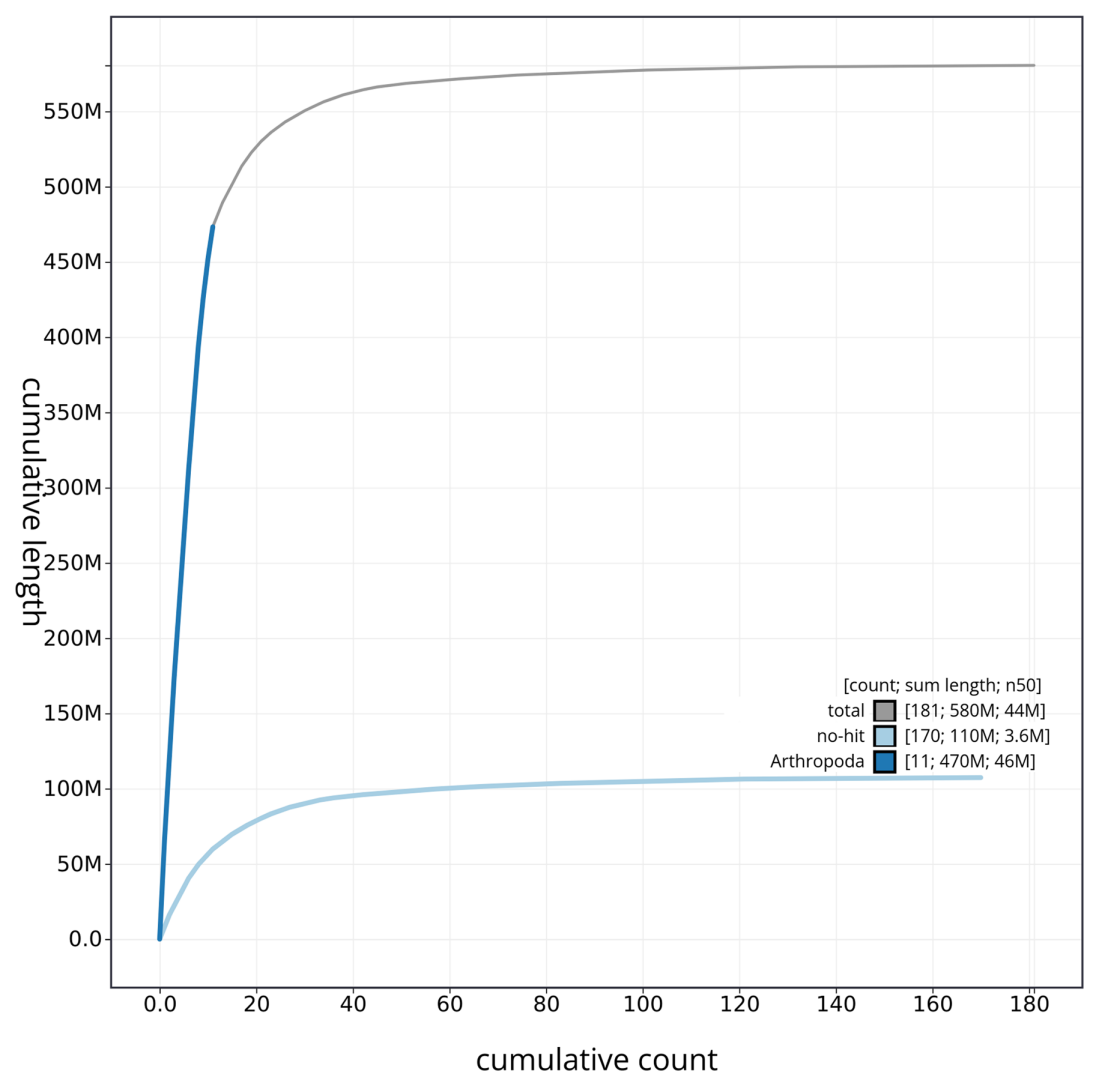
Genome assembly of *Geotrupes spiniger* icGeoSpin1.1: BlobToolKit cumulative sequence plot. The grey line shows cumulative length for all sequences. Coloured lines show cumulative lengths of sequences assigned to each phylum using the buscogenes taxrule. An interactive version of this figure is available at https://blobtoolkit.genomehubs.org/view/icGeoSpin1_1/dataset/icGeoSpin1_1/cumulative.

The snail plot in Figure 2 provides a summary of the assembly statistics, indicating the distribution of scaffold lengths and other assembly metrics. Figure 3 shows the distribution of scaffolds by GC proportion and coverage. Figure 4 presents a cumulative assembly plot, with separate curves representing different scaffold subsets assigned to various phyla, illustrating the completeness of the assembly.

**Figure 5.**
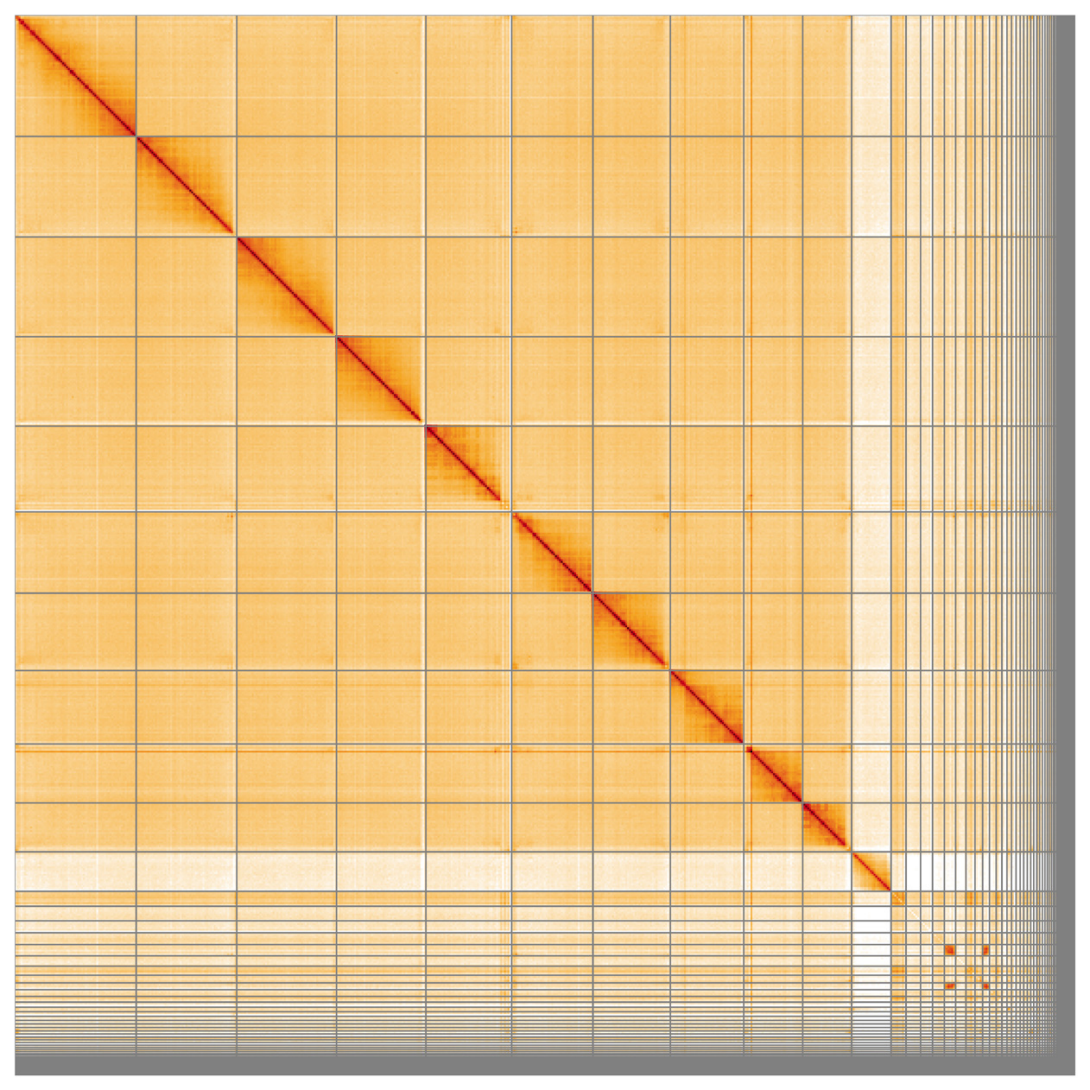
Genome assembly of *Geotrupes spiniger* icGeoSpin1.1: Hi-C contact map of the icGeoSpin1.1 assembly, visualised using HiGlass. Chromosomes are shown in order of size from left to right and top to bottom. An interactive version of this figure may be viewed at https://genome-note-higlass.tol.sanger.ac.uk/l/?d=I_GzqLAFQoyp36jIh5Gb8A.

Most of the assembly sequence (81.94%) was assigned to 12 chromosomal-level scaffolds, representing 10 autosomes and the X and Y sex chromosomes. These chromosome-level scaffolds, confirmed by the Hi-C data, are named in order of size ( Figure 5; Table 3). During manual curation, the sex chromosomes were assigned based on read coverage statistics and synteny to the genome assembly of *Cetonia aurata* (GCA_949128085.1).

**Table 3.** Chromosomal pseudomolecules in the genome assembly of *Geotrupes spiniger*, icGeoSpin1.

INSDC accession	Name	Length (Mb)	GC%
OY390731.1	1	65.61	34.0
OY390732.1	2	54.26	34.0
OY390733.1	3	53.93	34.0
OY390734.1	4	48.28	34.0
OY390735.1	5	46.22	34.0
OY390736.1	6	43.98	34.5
OY390737.1	7	41.81	34.5
OY390738.1	8	39.7	35.0
OY390739.1	9	31.82	36.0
OY390740.1	10	26.37	35.5
OY390741.1	X	21.3	33.0
OY390742.1	Y	2.47	28.5
OY390743.1	MT	0.02	21.0

While not fully phased, the assembly deposited is of one haplotype. Contigs corresponding to the second haplotype have also been deposited. The mitochondrial genome was also assembled and can be found as a contig within the multifasta file of the genome submission, and as a separate fasta file with accession OY390743.1.

The final assembly has a Quality Value (QV) of 62.0. The *k*-mer completeness is 74.46% for the primary assembly, 70.66% for the alternate haplotype, and 99.56% for the combined assemblies. BUSCO (v5.4.3) analysis using the endopterygota_odb10 reference set ( *n* = 2,124) indicated a completeness score of 99.5% (single = 98.7%, duplicated = 0.8%). The assembly achieves the Earth Biogenome Project (EBP) reference standard of 6.7.62. Other quality metrics are given in Table 2.

## Genome annotation report

The *Geotrupes spiniger* genome assembly (GCA_959613385.1) was annotated at the European Bioinformatics Institute (EBI) on Ensembl Rapid Release. The resulting annotation includes 21,852 transcribed mRNAs from 12,820 protein-coding and 1,582 non-coding genes ( Table 2; https://rapid.ensembl.org/Geotrupes_spiniger_GCA_959613385.1/Info/Index). The average transcript length is 12,472.01. There is an average of 1.52 coding transcripts per gene and 5.85 exons per transcript.

## Methods

### Sample acquisition and DNA barcoding

An adult male specimen of *Geotrupes spiniger* (specimen ID Ox000246, ToLID icGeoSpin1) was collected from dung on Wytham Farm, United Kingdom (latitude 51.78, longitude –1.31) on 2019-09-03. The specimen was collected and identified by František Sládeček (University of South Bohemia) and preserved on dry ice.

The initial identification was verified by an additional DNA barcoding process according to the framework developed by Twyford *et al*. (2024). A small sample was dissected from the specimens and stored in ethanol, while the remaining parts were shipped on dry ice to the Wellcome Sanger Institute (WSI). The tissue was lysed, the COI marker region was amplified by PCR, and amplicons were sequenced and compared to the BOLD database, confirming the species identification ( Crowley *et al.*, 2023). Following whole genome sequence generation, the relevant DNA barcode region was also used alongside the initial barcoding data for sample tracking at the WSI ( Twyford *et al.*, 2024). The standard operating procedures for Darwin Tree of Life barcoding have been deposited on protocols.io ( Beasley *et al.*, 2023).

### Nucleic acid extraction

The workflow for high molecular weight (HMW) DNA extraction at the Wellcome Sanger Institute (WSI) Tree of Life Core Laboratory includes a sequence of procedures: sample preparation and homogenisation, DNA extraction, fragmentation and purification. Detailed protocols are available on protocols.io ( Denton *et al.*, 2023b).

The icGeoSpin1 sample was prepared for DNA extraction by weighing and dissecting it on dry ice ( Jay *et al.*, 2023). Tissue from the thorax was homogenised using a PowerMasher II tissue disruptor ( Denton *et al.*, 2023a).

HMW DNA was extracted in the WSI Scientific Operations core using the Automated MagAttract v2 protocol ( Oatley *et al.*, 2023). The DNA was sheared into an average fragment size of 12–20 kb in a Megaruptor 3 system ( Bates *et al.*, 2023). Sheared DNA was purified by solid-phase reversible immobilisation, using AMPure PB beads to eliminate shorter fragments and concentrate the DNA ( Strickland *et al.*, 2023). The concentration of the sheared and purified DNA was assessed using a Nanodrop spectrophotometer and Qubit Fluorometer using the Qubit dsDNA High Sensitivity Assay kit. Fragment size distribution was evaluated by running the sample on the FemtoPulse system.

### Hi-C preparation

Tissue from the head tissue of the icGeoSpin1 sample was processed at the WSI Scientific Operations core, using the Arima-HiC v2 kit. Tissue (stored at –80 °C) was fixed, and the DNA crosslinked using a TC buffer with 22% formaldehyde. After crosslinking, the tissue was homogenised using the Diagnocine Power Masher-II and BioMasher-II tubes and pestles. Following the kit manufacturer's instructions, crosslinked DNA was digested using a restriction enzyme master mix. The 5’-overhangs were then filled in and labelled with biotinylated nucleotides and proximally ligated. An overnight incubation was carried out for enzymes to digest remaining proteins and for crosslinks to reverse. A clean up was performed with SPRIselect beads prior to library preparation.

### Library preparation and sequencing

Library preparation and sequencing were performed at the WSI Scientific Operations core. Pacific Biosciences HiFi circular consensus DNA sequencing libraries were prepared using the PacBio Express Template Preparation Kit v2.0 (Pacific Biosciences, California, USA) as per the manufacturer's instructions. The kit includes the reagents required for removal of single-strand overhangs, DNA damage repair, end repair/A-tailing, adapter ligation, and nuclease treatment. Library preparation also included a library purification step using AMPure PB beads (Pacific Biosciences, California, USA) and size selection step to remove templates shorter than 3 kb using AMPure PB modified SPRI. DNA concentration was quantified using the Qubit Fluorometer v2.0 and Qubit HS Assay Kit and the final library fragment size analysis was carried out using the Agilent Femto Pulse Automated Pulsed Field CE Instrument and gDNA 165kb gDNA and 55kb BAC analysis kit. Samples were sequenced using the Sequel IIe system (Pacific Biosciences, California, USA). The concentration of the library loaded onto the Sequel IIe was in the range 40–135 pM. The SMRT link software, a PacBio web-based end-to-end workflow manager, was used to set-up and monitor the run, as well as perform primary and secondary analysis of the data upon completion.

For Hi-C library preparation, DNA was fragmented to a size of 400 to 600 bp using a Covaris E220 sonicator. The DNA was then enriched, barcoded, and amplified using the NEBNext Ultra II DNA Library Prep Kit following manufacturers’ instructions. The Hi-C sequencing was performed using paired-end sequencing with a read length of 150 bp on an Illumina NovaSeq 6000 instrument.

### Genome assembly, curation and evaluation

***Assembly***

The HiFi reads were first assembled using Hifiasm ( Cheng *et al.*, 2021) with the --primary option. Haplotypic duplications were identified and removed using purge_dups ( Guan *et al.*, 2020). The Hi-C reads were mapped to the primary contigs using bwa-mem2 ( Vasimuddin *et al.*, 2019). The contigs were further scaffolded using the provided Hi-C data ( Rao *et al.*, 2014) in YaHS ( Zhou *et al.*, 2023) using the --break option for handling potential misassemblies. The scaffolded assemblies were evaluated using Gfastats ( Formenti *et al.*, 2022), BUSCO ( Manni *et al.*, 2021) and MERQURY.FK ( Rhie *et al.*, 2020).

The mitochondrial genome was assembled using MitoHiFi ( Uliano-Silva *et al.*, 2023), which runs MitoFinder ( Allio *et al.*, 2020) and uses these annotations to select the final mitochondrial contig and to ensure the general quality of the sequence.

***Assembly curation***

The assembly was decontaminated using the Assembly Screen for Cobionts and Contaminants (ASCC) pipeline (article in preparation). Manual curation was primarily conducted using PretextView ( Harry, 2022), with additional insights provided by JBrowse2 ( Diesh *et al.*, 2023) and HiGlass ( Kerpedjiev *et al.*, 2018). Scaffolds were visually inspected and corrected as described by Howe *et al*. (2021). Any identified contamination, missed joins, and mis-joins were corrected, and duplicate sequences were tagged and removed. The sex chromosomes were identified by read coverage statistics and synteny analysis. The curation process is documented at https://gitlab.com/wtsi-grit/rapid-curation (article in preparation).

***Evaluation of the final assembly***

The Merqury.FK tool ( Rhie *et al.*, 2020), run in a Singularity container ( Kurtzer *et al.*, 2017), was used to evaluate *k*-mer completeness and assembly quality for the primary and alternate haplotypes using the *k*-mer databases ( *k* = 31) that were computed prior to genome assembly. The analysis outputs included assembly QV scores and completeness statistics.

A Hi-C contact map was produced for the final version of the assembly. The Hi-C reads were aligned using bwa-mem2 ( Vasimuddin *et al.*, 2019) and the alignment files were combined using SAMtools ( Danecek *et al.*, 2021). The Hi-C alignments were converted into a contact map using BEDTools ( Quinlan & Hall, 2010) and the Cooler tool suite ( Abdennur & Mirny, 2020). The contact map is visualised in HiGlass ( Kerpedjiev *et al.*, 2018).

This genome was also analysed within the BlobToolKit environment ( Challis *et al.*, 2020) and BUSCO scores ( Manni *et al.*, 2021) were calculated.

Table 4 contains a list of relevant software tool versions and sources.

**Table 4.** Software tools: versions and sources.

Software tool	Version	Source
BEDTools	2.30.0	https://github.com/arq5x/bedtools2
BLAST	2.14.0	ftp://ftp.ncbi.nlm.nih.gov/blast/executables/blast+/
BlobToolKit	4.2.1	https://github.com/blobtoolkit/blobtoolkit
BUSCO	5.4.3	https://gitlab.com/ezlab/busco
bwa-mem2	2.2.1	https://github.com/bwa-mem2/bwa-mem2
Cooler	0.8.11	https://github.com/open2c/cooler
FastK	427104ea91c78c3b8b8b49f1a7d6bbeaa869ba1c	https://github.com/thegenemyers/FASTK
Gfastats	1.3.6	https://github.com/vgl-hub/gfastats
Hifiasm	0.16.1-r375	https://github.com/chhylp123/hifiasm
HiGlass	44086069ee7d4d3f6f3f0012569789ec138f42b84 aa44357826c0b6753eb28de	https://github.com/higlass/higlass
Merqury.FK	d00d98157618f4e8d1a9190026b19b471055b22e	https://github.com/thegenemyers/MERQURY.FK
MitoHiFi	3	https://github.com/marcelauliano/MitoHiFi
Nextflow	23.04.0-5857	https://github.com/nextflow-io/nextflow
PretextView	0.2.5	https://github.com/sanger-tol/PretextView
purge_dups	1.2.5	https://github.com/dfguan/purge_dups
samtools	1.16.1, 1.17, and 1.18	https://github.com/samtools/samtools
sanger-tol/ascc	-	https://github.com/sanger-tol/ascc
Singularity	3.9.0	https://github.com/sylabs/singularity
YaHS	1.2a.2	https://github.com/c-zhou/yahs

### Genome annotation

The Ensembl Genebuild annotation system ( Aken *et al.*, 2016) was used to generate annotation for the *Geotrupes spiniger* assembly (GCA_959613385.1) in Ensembl Rapid Release at the EBI. Annotation was created primarily through alignment of transcriptomic data to the genome, with gap filling via protein-to-genome alignments of a select set of proteins from UniProt ( UniProt Consortium, 2019).

### Wellcome Sanger Institute – Legal and Governance

The materials that have contributed to this genome note have been supplied by a Darwin Tree of Life Partner. The submission of materials by a Darwin Tree of Life Partner is subject to the **‘Darwin Tree of Life Project Sampling Code of Practice’**, which can be found in full on the Darwin Tree of Life website here. By agreeing with and signing up to the Sampling Code of Practice, the Darwin Tree of Life Partner agrees they will meet the legal and ethical requirements and standards set out within this document in respect of all samples acquired for, and supplied to, the Darwin Tree of Life Project.

Further, the Wellcome Sanger Institute employs a process whereby due diligence is carried out proportionate to the nature of the materials themselves, and the circumstances under which they have been/are to be collected and provided for use. The purpose of this is to address and mitigate any potential legal and/or ethical implications of receipt and use of the materials as part of the research project, and to ensure that in doing so we align with best practice wherever possible. The overarching areas of consideration are:

•    Ethical review of provenance and sourcing of the material

•    Legality of collection, transfer and use (national and international)

Each transfer of samples is further undertaken according to a Research Collaboration Agreement or Material Transfer Agreement entered into by the Darwin Tree of Life Partner, Genome Research Limited (operating as the Wellcome Sanger Institute), and in some circumstances other Darwin Tree of Life collaborators.

## Data Availability

European Nucleotide Archive: Geotrupes spiniger. Accession number PRJEB64062; https://identifiers.org/ena.embl/PRJEB64062. The genome sequence is released openly for reuse. The *Geotrupes spiniger* genome sequencing initiative is part of the Darwin Tree of Life (DToL) project. All raw sequence data and the assembly have been deposited in INSDC databases. Raw data and assembly accession identifiers are reported in Table 1 and Table 2.

## Author information

Members of the University of Oxford and Wytham Woods Genome Acquisition Lab are listed here: https://doi.org/10.5281/zenodo.12157525.

Members of the Darwin Tree of Life Barcoding collective are listed here: https://doi.org/10.5281/zenodo.12158331.

Members of the Wellcome Sanger Institute Tree of Life Management, Samples and Laboratory team are listed here: https://doi.org/10.5281/zenodo.12162482.

Members of Wellcome Sanger Institute Scientific Operations: Sequencing Operations are listed here: https://doi.org/10.5281/zenodo.12165051.

Members of the Wellcome Sanger Institute Tree of Life Core Informatics team are listed here: https://doi.org/10.5281/zenodo.12160324.

Members of the Tree of Life Core Informatics collective are listed here: https://doi.org/10.5281/zenodo.12205391.

Members of the Darwin Tree of Life Consortium are listed here: https://doi.org/10.5281/zenodo.4783558.
