# Supplementary material for: The genome sequence of a dung beetle, Geotrupes spiniger (Marsham, 1802)
Source: Wellcome Open Res. 2025 Oct 1;10:71. Originally published 2025 Feb 13. [Version 2] doi: 10.12688/wellcomeopenres.23705.2 (PMC12648035; doi:10.12688/wellcomeopenres.23705.2)
Supplement: Peer review history

# Reviewer report: Reviewer response for version 2

**DOI:** 10.21956/wellcomeopenres.27578.r140268

**Published:** 2025-11-25

**Authors:** Chenyang Cai

**Affiliations:** 1 Nanjing Institute of Geology and Palaeontology, Beijing, Beijing, China

This manuscript presents a high-quality, chromosome-level genome assembly of the dung beetle *Geotrupes spiniger*, generated as part of the Darwin Tree of Life project. The assembly exhibits commendable contiguity and completeness, with 81.94% of the sequence scaffolded into 12 chromosomal pseudomolecules, including sex chromosomes. The study provides a valuable genomic resource for an ecologically significant species, supporting future research in insect evolution, dung beetle biology, and ecosystem functioning. The manuscript is generally well-structured and the data appear robust.

 One suggestion: it would be beneficial to include standard metrics of assembly and annotation completeness, such as BUSCO score (99.5% ) in the Abstract.

Are sufficient details of methods and materials provided to allow replication by others?

Yes

Is the rationale for creating the dataset(s) clearly described?

Yes

Are the datasets clearly presented in a useable and accessible format?

Yes

Are the protocols appropriate and is the work technically sound?

Yes

Reviewer Expertise:

Beetle genomics

I confirm that I have read this submission and believe that I have an appropriate level of expertise to confirm that it is of an acceptable scientific standard.

# Reviewer report: Reviewer response for version 1

**DOI:** 10.21956/wellcomeopenres.26153.r118866

**Published:** 2025-03-13

**Authors:** Lili Ren

**Affiliations:** 1 Beijing Forestry University, Beijing, China

The study performed genome sequencing and assembly of *Geotrupes spiniger*, obtaining a chromosome-level genome sequence. The findings provide valuable insights into the biological mechanisms of this species. However, I have the following concerns regarding the methods and results presented in the manuscript:

1. The study assembled the X and Y chromosomes of *G. spiniger *and conducted a synteny analysis with *Cetonia aurata*. However, the sequencing was based on a single male individual. I suggest incorporating resequencing data from a female individual to assist in Hi-C assembly, thereby enhancing the reliability of the results.
2. The GC content of *G. spiniger* is reported as 33.4%. However, some data in Figures 2 and 3 show a higher GC content. I suggest verifying the reliability of these data and assessing whether they may be affected by potential contamination.
3. The study obtained 12 chromosome-level scaffolds through Hi-C assembly, including 10 autosomes and the X and Y sex chromosomes. Please label these in Figure 5. Additionally, I recommend manually adjusting the Hi-C assembly results, particularly the data in the lower right corner of Figure 5, to increase the anchoring rate.

Are sufficient details of methods and materials provided to allow replication by others?

Yes

Is the rationale for creating the dataset(s) clearly described?

Yes

Are the datasets clearly presented in a useable and accessible format?

Yes

Are the protocols appropriate and is the work technically sound?

Yes

Reviewer Expertise:

entomology, forest pest

I confirm that I have read this submission and believe that I have an appropriate level of expertise to confirm that it is of an acceptable scientific standard, however I have significant reservations, as outlined above.

# Response

**Published:** 2025-09-24

**Authors:** Tree of Life Team Sanger

**Affiliations:** Wellcome Trust Sanger Institute, UK

*We thank you for review and respond to your comments below:*

1. The study assembled the X and Y chromosomes of *G. spiniger *and conducted a synteny analysis with *Cetonia aurata*. However, the sequencing was based on a single male individual. I suggest incorporating resequencing data from a female individual to assist in Hi-C assembly, thereby enhancing the reliability of the results.

*Response: The Hi-C data were from the same individual as used for the PacBio sequencing, which is the most reliable way to scaffold an assembly, and also allows for phasing of the chromosomes. The advantage of using the heterogametic sex is that both sex chromosomes are sequenced. The goals of the Darwin Tree of Life project do not include resequencing of species.*

1. The GC content of *G. spiniger* is reported as 33.4%. However, some data in Figures 2 and 3 show a higher GC content. I suggest verifying the reliability of these data and assessing whether they may be affected by potential contamination.

*Response: The assembly has been checked for contamination, and the results have been validated. The GC content of the chromosomal scaffolds vary from 33% to 36%. Scaffolds that fall outside of this range have no BUSCO hits. Please see the Tree of Life assembly pipelines here:* https://pipelines.tol.sanger.ac.uk/. *We have included this link in the Data availability section of the article.*  

1. The study obtained 12 chromosome-level scaffolds through Hi-C assembly, including 10 autosomes and the X and Y sex chromosomes. Please label these in Figure 5. Additionally, I recommend manually adjusting the Hi-C assembly results, particularly the data in the lower right corner of Figure 5, to increase the anchoring rate.

*Response: In version 2 of this data note we have included labels on Figure 5 (note that the Y chromosome is too tiny to label). This assembly has already been manually curated by expert genome finishers. The contigs at the end of the assembly are repeat-rich and cannot be placed.*

# Reviewer report: Reviewer response for version 1

**DOI:** 10.21956/wellcomeopenres.26153.r118874

**Published:** 2025-03-04

**Authors:** Lindsey C Perkin

**Affiliations:** 1 USDA-ARS, Southern Plains Agricultural Research Center, College Station, USA

The authors present a very nice genome and mitochondrial genome for the dung beetle.

 The authors sequence a male beetle with PacBio Hifi and Hi-C scffolding - methodologies are well described and considered the gold standard for the field. They achieve high sequence depth and show a nice continuous reference-quality genome. 

 The authors also annotate using the Ensemle pipeline.

 The manuscript should be indexed.

Are sufficient details of methods and materials provided to allow replication by others?

Yes

Is the rationale for creating the dataset(s) clearly described?

Yes

Are the datasets clearly presented in a useable and accessible format?

Yes

Are the protocols appropriate and is the work technically sound?

Yes

Reviewer Expertise:

Genomics and transcriptomics

I confirm that I have read this submission and believe that I have an appropriate level of expertise to confirm that it is of an acceptable scientific standard.
